# Evaluating the Efficacy and Cost-Effectiveness of Plasmapheresis and Intravenous Immunoglobulin in Acute Guillain-Barre Syndrome Management in Emergency Departments

**DOI:** 10.7759/cureus.72595

**Published:** 2024-10-29

**Authors:** Waseem Ullah, Haider Ali, Yasir Muhammad, Naqeeb Ullah, Iqra Khalil, Sohail Ahmad, Obaid Ullah, Amjad Ali, Muhammad Younas Ali, Awais Manan, Sundas Safdar, Hanifullah Hanfi

**Affiliations:** 1 Acute Medicine, Russells Hall Hospital, Dudley, GBR; 2 Internal Medicine (Specialty Group), Khyber Teaching Hospital, Peshawar, PAK; 3 General Medicine, Syed Medical Complex, Sialkot, PAK; 4 Internal Medicine, Lady Reading Hospital, Peshawar, PAK; 5 Medicine, Rehman Medical Institute, Peshawar, PAK; 6 Medicine, Sunderland Royal Hospital, Sunderland, GBR; 7 Internal Medicine, Rehman Medical Institute, Peshawar, PAK; 8 Medicine and Surgery, Gajju Khan Medical College, Nowshera, PAK; 9 General Physician, Bangash Clinic Doaba, Kohat, PAK; 10 Radiology, Hayatabad Medical Complex, Peshawar, PAK; 11 Internal Medicine, Mardan Medical Complex, Mardan, PAK

**Keywords:** cost-effectiveness, emergency department, guillain-barre syndrome, intravenous immunoglobulin, plasmapheresis

## Abstract

Background: Guillain-Barre Syndrome (GBS) is a severe autoimmune neuropathy that has to be treated quickly and efficiently in emergency situations, when both cost and effectiveness are vital.

Objective: To compare the clinical outcomes and cost-effectiveness of plasmapheresis and intravenous immunoglobulin (IVIG) in managing acute GBS in emergency departments.

Methodology: A prospective observational study conducted from January to December 2023 evaluated the treatment of adults with acute GBS using IVIG or plasmapheresis. Cost analysis was done in conjunction with clinical outcomes assessment, such as improvement in motor function, length of recovery, and length of hospital stay. Each therapy group consisted of 39 individuals, and data were gathered via patient evaluations and medical records. Through statistical analysis, the two regimens' costs and results were compared.

Results: Plasmapheresis proved significantly more cost-effective than IVIG, with an average treatment cost of 295,000 Pakistani rupees (PKR) per patient versus 587,000 PKR for IVIG. Plasmapheresis showed a greater mean improvement in motor function (Glasgow Coma Scale (GCS) score change of 5.93 ± 2.17) compared to IVIG (4.98 ± 2.34), though the difference was not statistically significant (p=0.091). Recovery time was slightly shorter with plasmapheresis, averaging 8.26 ± 1.53 weeks versus 8.74 ± 1.87 weeks for IVIG (p=0.282). Hospital length of stay was marginally reduced with plasmapheresis (12.16 ± 3.28 days) compared to IVIG (12.95 ± 3.64 days, p=0.302). The time to reach maximum improvement was also shorter with plasmapheresis (6.51 ± 1.24 weeks) compared to IVIG (7.08 ± 1.32 weeks, p=0.260). Additionally, plasmapheresis had a lower complication rate, with 4 out of 39 patients (10.25%) experiencing complications, compared to 8 out of 39 patients (20.51%) with IVIG.

Conclusion: Plasmapheresis is more cost-effective than IVIG for acute GBS management in emergency departments, offering similar or potentially superior clinical outcomes at a lower cost.

## Introduction

Guillain-Barre Syndrome (GBS) is an autoimmune neuropathy that progresses quickly and often manifests as a medical emergency [1,2]. GBS is characterized by symmetrical limb weakness, autonomic dysfunction, and sensory abnormalities. It may cause life-threatening consequences. Hence, it is essential to get fast and efficient treatment [3]. Intravenous immunoglobulin (IVIG) and plasmapheresis are the two main treatments for the acute management of GBS that have shown promise in clinical studies [4]. But more work has to be done to assess these treatments critically in terms of their cost-effectiveness as well as clinical efficacy, especially in the high-stress setting of emergency rooms [5].

Pathogenic antibodies are extracted from the circulation during plasmapheresis, and this procedure is thought to have a role in the demyelination phase of GBS [6]. It has been shown that this process, which is usually spread out across multiple sessions, improves motor function and shortens the recovery period [7]. On the other hand, IVIG offers a wide range of immunomodulatory effects and may reduce the autoimmune reaction by infusing combined immunoglobulins from healthy donors [8]. The patient's condition, availability, and cost of care are some of the elements that often influence the decision between these therapies [9].

The need for quick action and resource optimization complicates the decision-making process for GBS management in emergency departments [10]. Treatment decisions may be influenced by the unique logistical and financial issues that IVIG and plasmapheresis both bring [11]. Although professional guidelines provide broad suggestions, little is known about how these treatments fare in actual emergency situations, when finding quick and affordable remedies is crucial [12]. By methodically comparing the effectiveness and financial implications of plasmapheresis and IVIG in the treatment of acute GBS in emergency rooms, this study aims to close this gap.

Research objective

Objective of this study was to compare the clinical outcomes, including motor function improvement, recovery times, and complication rates, as well as the cost-effectiveness of plasmapheresis and IVIG in managing acute GBS within emergency departments.

## Materials and methods

Ethical approval

The Institutional Review Board (IRB) at Khyber Medical College issued approval (ERC/DME/MTI/KTH-22/019) before conducting this study. Consent was obtained from all participants or their legal guardians before being included in the research. Every step followed the moral standards for doing research on human beings.

Study design and setting

A prospective observational study was conducted at Khyber Teaching Hospital from January to December 2023 to evaluate the treatment of adults with acute GBS using IVIG or plasmapheresis. The efficacy and cost-effectiveness of plasmapheresis vs IVIG in the treatment of acute GBS were evaluated in this research using a prospective, observational approach. 

Inclusion and exclusion criteria

The study population included people who met the following criteria: they had to be diagnosed with acute GBS based on clinical and diagnostic criteria, be older than 18 years old, and begin IVIG or plasmapheresis therapy within four weeks of the beginning of symptoms. Informed permission was another essential need for inclusion. The exclusion criteria included people with secondary GBS, serious co-occurring diseases that may affect the outcomes, people receiving concurrent medications that weren't included in the research, people who were pregnant or nursing, people with insufficient medical records, and those who stopped their treatment early.

Sample size

We estimated that each group would consist of 39 patients to examine the effectiveness and cost-benefits of IVIG versus plasmapheresis in treating acute GBS. The trial included a total of 78 individuals, with 39 patients receiving IVIG and 39 receiving plasmapheresis. A convenience sampling technique was employed to select participants for this study. This sample size provides sufficient statistical power to detect significant differences in both clinical outcomes and costs between the two interventions. Similar sample sizes have been used in a recent study, which utilized 35 patients per group to evaluate treatment effectiveness in GBS management. This supports the adequacy of our chosen sample size for reliable analysis [13].

Data collection

Data were gathered through three primary methods: direct patient assessments, follow-up evaluations, and reviews of medical records. To specifically assess recovery time, follow-up evaluations were conducted at predefined intervals: one week, two weeks, four weeks, and eight weeks post-discharge. During these follow-up visits, patients were evaluated for improvements in motor function, assessed using the modified Medical Research Council (MRC) scale, and queried about their recovery experiences, including any residual symptoms or complications. Medical records were reviewed to corroborate self-reported recovery times and ensure accuracy. Important factors included demographic information, clinical outcomes (such as improved motor function and recovery times), and financial data related to each treatment modality. Standardized instruments and procedures were employed throughout the data collection process to guarantee accuracy and consistency.

Treatment protocols

The decision to treat patients with either IVIG or plasmapheresis was based on a standardized protocol developed by the treating physicians, which considered clinical severity assessed through the Glasgow Coma Scale (GCS) and the patient's specific medical history. The GCS is a clinical scale used to assess a patient's level of consciousness by evaluating three aspects: eye, verbal, and motor responses, with scores ranging from 3 to 15, where a higher score indicates better neurological function. Patients presenting with moderate to severe symptoms, characterized by significant muscle weakness or respiratory involvement, were prioritized for plasmapheresis, while those with mild symptoms or contraindications for plasmapheresis were treated with IVIG to minimize selection bias and ensure appropriate use of each treatment modality. Cost analysis was conducted alongside clinical outcomes assessment, including improvement in motor function, length of recovery, and length of hospital stay. Each therapy group consisted of 39 individuals, and data were gathered through patient evaluations and medical records. 

Patients receiving IVIG were treated with a dose of 0.4 g/kg per day over a total of five days, culminating in a cumulative dose of 2 g/kg. For those undergoing plasmapheresis, the procedure involved four to five sessions using a standard double filtration technique, with each session lasting approximately two to three hours and removing about 1.5 to 2 times the patient’s total blood volume. Plasma was replaced with an appropriate substitution fluid, such as albumin or normal saline, to maintain blood volume and protein levels. These standardized treatment protocols ensured consistency in the management of patients across both groups.

Statistical analysis

In our statistical analysis, we employed both descriptive and inferential statistics to evaluate the differences between the two treatment groups (IVIG and plasmapheresis). Continuous variables, including age, motor function improvement, recovery time, and length of hospital stay, were analyzed using independent t-tests, while categorical variables, such as the incidence of complications and rates of severe recovery, were assessed using chi-square tests. A p-value of less than 0.05 was considered statistically significant. Recognizing the inherent limitations of a relatively small sample size (n=39 per group), we approached the interpretation of our results with caution. Although some outcomes did not achieve statistical significance (p=0.091 for motor function improvement), we also assessed the clinical relevance of these findings. Effect sizes were calculated to better understand the practical implications of our results, emphasizing that non-significant p-values may still indicate clinically meaningful differences that warrant further investigation.

## Results

The demographic details of research participants undergoing IVIG (n=39) vs plasmapheresis (n=39) are shown in Table 1. The plasmapheresis group had an average age of 45.27 ± 12.41 years, whereas the IVIG group had an average age of 46.13 ± 11.89 years. For plasmapheresis, the age distribution was 8 (20.51%) in the 18-30 age range, 16 (41.03%) in the 31-50 age range, and 15 (38.46%) beyond 51 years; for IVIG, it was 7 (17.95%), 15 (38.46%), and 17 (43.59%). In the plasmapheresis group, there were 22 males (56.41%) and 17 females (43.59%), whereas in the IVIG group, there were 20 males (51.28%) and 19 females (48.72%). The following coexisting conditions were identified: for plasmapheresis, there were nine patients (23.08%) with hypertension and six patients (15.38%) with diabetes mellitus; for IVIG, there were eight patients (20.51%) with hypertension and seven patients (17.95%) with diabetes mellitus.

According to GCS scores, the initial severity for plasmapheresis was mild in 12 patients (30.77%), moderate in 21 patients (53.85%), and severe in 6 patients (15.38%); likewise, for IVIG, the initial severity was mild in 11 patients (28.21%), moderate in 20 patients (51.28%), and severe in 8 patients (20.51%). A statistical comparison using chi-square tests indicated that while the overall distribution of severity categories was similar between groups, there was a slightly higher proportion of severe cases in the IVIG group (20.51% vs. 15.38%, p=0.378). This suggests that the initial severity of cases may influence treatment outcomes.

A detailed analysis of baseline motor function scores revealed a mean of 12.35 ± 3.43 for plasmapheresis and 11.93 ± 3.65 for IVIG. An independent t-test indicated that this difference was not statistically significant (t=-0.612, p=0.543), confirming that baseline motor function was comparable between groups. Before receiving therapy, the average length of time for symptoms was 2.88 ± 1.07 weeks for plasmapheresis and 3.01 ± 1.23 weeks for IVIG as shown in Table 1.

**Table 1:** Demographic characteristics of study participants GCS: Glasgow Coma Scale; IVIG: Intravenous immunoglobulin

Characteristic		Plasmapheresis (n=39)	IVIG (n=39)	P-value
Age (years)	18-30	8 (20.51%)	7 (17.95%)	-
	31-50	16 (41.03%)	15 (38.46%)	-
	51+	15 (38.46%)	17 (43.59%)	-
	Mean ± SD	45.27 ± 12.41	46.13 ± 11.89	-
Gender	Male	22 (56.41%)	20 (51.28%)	-
	Female	17 (43.59%)	19 (48.72%)	-
Comorbidities	Hypertension	9 (23.08%)	8 (20.51%)	-
	Diabetes Mellitus	6 (15.38%)	7 (17.95%)	-
	Cardiovascular Disease	4 (10.26%)	5 (12.82%)	-
Initial Severity (GCS Score)	Mild (GCS Score 8-12)	12 (30.77%)	11 (28.21%)	0.378
	Moderate (GCS Score 4-7)	21 (53.85%)	20 (51.28%)	
	Severe (GCS Score <4)	6 (15.38%)	8 (20.51%)	
Duration of Symptoms Before Treatment (weeks)	Mean ± SD	2.88 ± 1.07	3.01 ± 1.23	-
Baseline Motor Function Score (GCS)	Mean ± SD	12.35 ± 3.43	11.93 ± 3.65	0.543

The comparative studies of clinical outcomes between IVIG (n=39) and plasmapheresis (n=39) are shown in Table 2. Although this difference was not statistically significant (p=0.232), the incidence of complications was lower in the plasmapheresis group, with four patients (10.25%), than in the IVIG group, with eight patients (20.51%). In the plasmapheresis group, complications included two patients (5.13%) who experienced hypotension and two patients (5.13%) with electrolyte imbalances, while no serious complications were reported. In the IVIG group, one patient (2.56%) experienced anaphylaxis, three patients (7.69%) had allergic reactions, two patients (5.13%) developed renal dysfunction, and one patient (2.56%) experienced thromboembolic events, along with three patients (7.69%) suffering from headaches. With no discernible difference (p=0.469), nine patients (23.08%) receiving plasmapheresis and seven patients (18.18%) receiving IVIG had severe recovery, which is defined as a return to normal GCS scores. Compared to IVIG (4.98 ± 2.34), plasmapheresis demonstrated a larger mean improvement in motor function (GCS score change of 5.93 ± 2.17); nevertheless, this difference approached but did not achieve statistical significance (p=0.091). While IVIG (8.74 ± 1.87 weeks) required a longer recovery period than plasmapheresis (8.26 ± 1.53 weeks), this difference was not statistically significant (p=0.282). Additionally, there was a little variation in the duration of hospital stay between IVIG (12.95 ± 3.64 days) and plasmapheresis (12.16 ± 3.28 days), with no significant difference (p=0.302). Despite not being statistically significant (p=0.260), plasmapheresis took less time to obtain maximal improvement (6.51 ± 1.24 weeks) than IVIG (7.08 ± 1.32 weeks). There was no significant difference (p=0.287) in the time to treatment initiation between IVIG (3.97 ± 1.48 days) and plasmapheresis (3.43 ± 1.21 days).

**Table 2:** Comparative analyses of clinical outcomes between plasmapheresis and IVIG GCS: Glasgow Coma Scale; IVIG: Intravenous immunoglobulin

Outcome	Plasmapheresis (n=39)	IVIG (n=39)	P-value
Rate of Complications (n (%))	4 (10.25%)	8 (20.51%)	0.232
Serious Complications	0 (0%)	1 (2.56%) (Anaphylaxis)	
Hypotension	2 (5.13%)	-	
Electrolyte Imbalance	2 (5.13%)	-	
Allergic Reactions	-	3 (7.69%)	
Renal Dysfunction	-	2 (5.13%)	
Thromboembolic Events	-	1 (2.56%)	
Headaches	-	3 (7.69%)	
Patients with Severe Recovery (n (%))	9 (23.08%)	7 (18.18%)	0.469
Improvement in Motor Function (GCS Score Change)	5.93 ± 2.17	4.98 ± 2.34	0.091
Recovery Time (Weeks)	8.26 ± 1.53	8.74 ± 1.87	0.282
Hospital Length of Stay (Days)	12.16 ± 3.28	12.95 ± 3.64	0.302
Duration to Reach Maximum Improvement (Weeks)	6.51 ± 1.24	7.08 ± 1.32	0.26
Time to Treatment (dDys)	3.43 ± 1.21	3.97 ± 1.48	0.287

When treating acute GBS, Table 3 compares the clinical results and expenses of IVIG (n=39) vs plasmapheresis (n= 39). Compared to IVIG, which affected eight patients (20.51%), plasmapheresis resulted in four patients (10.25%) with a decreased risk of problems. Nine patients (23.08%) getting plasmapheresis had severe recovery, defined as a return to normal GCS scores, compared to seven patients (18.18%) receiving IVIG. The mean improvement in motor function was higher with plasmapheresis (5.93 ± 2.17 GCS score change) than IVIG (4.98 ± 2.34). Using plasmapheresis, the average recovery period was 8.26 ± 1.53 weeks, whereas IVIG required 8.74 ± 1.87 weeks. Additionally, plasmapheresis patients' hospital stays lasted 12.16 ± 3.28 days, which is somewhat less than IVIG patients' (12.95 ± 3.64 days) stays. It took 6.51 ± 1.24 weeks with plasmapheresis and 7.08 ± 1.32 weeks with IVIG to get the greatest improvement. The start of therapy took somewhat less time for IVIG (3.97 ± 1.48 days) than for plasmapheresis (3.43 ± 1.21 days). Last but not least, plasmapheresis costs far less at 295,000 Pakistani rupees (PKR) than IVIG, which costs 587,000 PKR.

**Table 3:** Clinical outcomes and cost of plasmapheresis versus IVIG ^*^: $1,062; ^#^: $2,113 GCS: Glasgow Coma Scale; IVIG: Intravenous immunoglobulin; PKR: Pakistani rupees

Outcome	Plasmapheresis (n=39)	IVIG (n=39)
Rate of Complications (n (%))	4 (10.25)	8 (20.51)
Patients with Severe Recovery (GCS Score Return to Normal) (n (%))	9 (23.08)	7 (18.18)
Improvement in Motor Function (GCS Score Change) (Mean ± SD)	5.93 ± 2.17	4.98 ± 2.34
Recovery Time (Weeks) (Mean ± SD)	8.26 ± 1.53	8.74 ± 1.87
Hospital Length of Stay (Days) (Mean ± SD)	12.16 ± 3.28	12.95 ± 3.64
Duration to Reach Maximum Improvement (Weeks) (Mean ± SD)	6.51 ± 1.24	7.08 ± 1.32
Time to Treatment (Days) (Mean ± SD)	3.43 ± 1.21	3.97 ± 1.48
Cost (PKR)	295,000^*^	587,000^#^

In our comparative analysis, we observed trends favoring plasmapheresis in several key outcomes, including motor function improvement (5.93 ± 2.17 GCS score change for plasmapheresis vs. 4.98 ± 2.34 for IVIG, p=0.091) and shorter recovery times (8.26 ± 1.53 weeks for plasmapheresis vs. 8.74 ± 1.87 weeks for IVIG, p=0.282). While these differences did not reach statistical significance, they suggest a potential advantage for plasmapheresis in improving clinical outcomes.

Furthermore, the rate of complications was lower in the plasmapheresis group (10.25%) compared to the IVIG group (20.51%, p=0.232), indicating a trend towards greater safety with plasmapheresis. These findings, though not statistically significant, underscore the importance of considering clinical significance alongside statistical results. The observed trends provide valuable insights for clinicians when choosing treatment options for patients with acute GBS, particularly in light of the substantial cost difference between the two therapies.

A comparison of the cost-effectiveness of IVIG (n=39) and plasmapheresis (n=39) for the treatment of acute GBS is shown in Table 4. Compared to IVIG, which has a treatment cost per patient of 587,000 PKR, plasmapheresis has a total treatment cost per patient of 295,000 PKR. Although there was a cost difference, the mean improvement in motor function with plasmapheresis was higher than with IVIG, with a GCS score change of 5.93 as opposed to 4.98. Additionally, plasmapheresis recovery took an average of 8.26 weeks as opposed to 8.74 weeks for IVIG.

**Table 4:** Cost-effectiveness analysis PKR: Pakistani rupees; GCS: Glasgow Coma Scale; IVIG: Intravenous immunoglobulin

Parameter	Plasmapheresis (n=39)	IVIG (n=39)
Total Treatment Cost (PKR)	11,505,000	22,833,000
Cost per Patient (PKR)	295,000	587,000
Improvement in Motor Function (GCS Score Change)	5.93	4.98
Recovery Time (Weeks)	8.26	8.74

## Discussion

The results of this investigation provide significant new information on the relative cost- and effectiveness of IVIG and plasmapheresis in the treatment of acute GBS in emergency rooms. Our findings show that IVIG, which costs 587,000 PKR, is much more expensive than plasmapheresis, which has a mean cost of 295,000 PKR. This discrepancy in costs is consistent with other research that has emphasized the financial benefits of plasmapheresis in the management of GBS [6,14]. According to research by Nandeesha et al., plasmapheresis is typically less costly than IVIG and has similar effectiveness in terms of improving patient outcomes [15].

In terms of clinical results, our research revealed that plasmapheresis (mean change of 5.93) produced a higher improvement in motor function than IVIG (mean change of 4.98), as shown by improvements in GCS scores. This conclusion is in line with other studies that suggested, albeit the differences were not always statistically significant, that plasmapheresis could provide superior functional outcomes than IVIG [16,17]. It is important to note, however, that our data did not reveal statistically significant differences in hospital length of stay or recovery time between the two therapies; the recovery time for IVIG was 8.74 weeks, while plasmapheresis was 8.26 weeks. These results are in line with earlier research, which found no discernible variation in recovery periods between the two therapies [18,19].

IVIG had a greater incidence of complications (20.51%) than did plasmapheresis, with a rate of 10.25%. This difference implies that plasmapheresis may be linked with a decreased risk of problems compared to IVIG, even if it did not approach statistical significance (p=0.232). This data corroborates the results of research by Ortiz et al., which showed that plasmapheresis had fewer side effects than IVIG [20]. Additionally, plasmapheresis required less time to obtain maximal improvement (6.51 weeks) than IVIG (7.08 weeks), suggesting a possible faster response to therapy (although this difference was not statistically significant).

All things considered, our research offers insightful information on the relative merits and financial consequences of IVIG vs plasmapheresis in the treatment of acute GBS. Although the two therapies' clinical results were comparable, plasmapheresis showed a distinct benefit in terms of cost-effectiveness and possibly fewer side effects. These results are in line with previous research and highlight the need to consider clinical and financial aspects when choosing a GBS therapy in emergency situations.

Study limitations and strengths

One of the key strengths of our study is the standardized treatment protocols employed, which minimized variability and enhanced the reliability of the results. The prospective observational design allowed for real-time data collection and assessment of patient outcomes, making our findings highly relevant to clinical practice. Our comprehensive data analysis included both clinical and economic outcomes, providing a holistic view of the treatment options.

Although the study was conducted at a single institution, this focused setting allowed for standardized treatment and consistent data collection, enhancing the internal validity of the findings. Future research could expand upon our work by including diverse patient populations and healthcare settings, which would strengthen the generalizability of the results. Despite these limitations, our findings contribute meaningfully to the growing body of evidence on the treatment of acute GBS and underscore the importance of considering both statistical and clinical significance in evaluating therapeutic options.

We also recognize that non-significant findings should not be dismissed; they can inform clinical practice and guide future research. The observed trends, such as improved motor function and shorter recovery times with plasmapheresis, suggest the need for larger, multi-center studies to validate these results and further explore the clinical implications.

## Conclusions

Our research shows that plasmapheresis, with a far lower treatment cost and a possible decreased risk of complications, is more cost-effective than IVIG for treating acute GBS in emergency rooms. While the clinical results of both therapies are comparable in terms of improved motor function and faster recovery periods, plasmapheresis offers a clear benefit in terms of cost-effectiveness and a faster route to maximal improvement. These results provide evidence in favor of plasmapheresis as the treatment of choice for acute GBS when cost-effectiveness and clinical efficacy are taken into account.
